# Fast, axis-agnostic, dynamically summarized storage and retrieval for mass spectrometry data

**DOI:** 10.1371/journal.pone.0188059

**Published:** 2017-11-15

**Authors:** Kyle Handy, Jebediah Rosen, André Gillan, Rob Smith

**Affiliations:** Department of Computer Science, University of Montana, Missoula, Montana, United States of America; Pacific Northwest National Laboratory, UNITED STATES

## Abstract

Mass spectrometry, a popular technique for elucidating the molecular contents of experimental samples, creates data sets comprised of millions of three-dimensional (m/z, retention time, intensity) data points that correspond to the types and quantities of analyzed molecules. Open and commercial MS data formats are arranged by retention time, creating latency when accessing data across multiple m/z. Existing MS storage and retrieval methods have been developed to overcome the limitations of retention time-based data formats, but do not provide certain features such as dynamic summarization and storage and retrieval of point meta-data (such as signal cluster membership), precluding efficient viewing applications and certain data-processing approaches. This manuscript describes MzTree, a spatial database designed to provide real-time storage and retrieval of dynamically summarized standard and augmented MS data with fast performance in both m/z and RT directions. Performance is reported on real data with comparisons against related published retrieval systems.

## Introduction

Liquid chromatography-mass spectrometry is a popular technique often used for high-throughput protein, lipid, and metabolite experimental quantification and identification [1]. Quantification and identification of molecules requires the computational analysis of raw mass spectrometer output [2], files consisting of millions of three-dimensional points with mass-to-charge (m/z), retention time (RT), and intensity axes. Standard non-proprietary file formats for mass spectrometry (MS) data are *mzML* [3] and *mzXML* [4]. Like its proprietary counterparts, mzML is arranged by RT scans. RT-centric data formats make time-efficient data processing a challenge, as it requires the traversal of MS data scan by scan, making it impossible to quickly extract regional windows bounded by an m/z interval over several RT scans. This limitation precludes certain MS data processing techniques that require regional data queries that would be computationally intensive with RT-centric formats such as native mzML.

Recognizing the limitations of native mzML, the community has developed several storage and retrieval systems for MS data, such as mz5 [5] and mzDB [6]. mz5 is a queryable MS data store based on the HDF5 database system and provides faster access than XML-based mzML, but still requires iteration through each relevant scan in order to retrieve the subset of individual points in the requested window.

mzDB provides fast, axis-agnostic access to MS data for small queries. However, mzDB is designed to return all points within query bounds without intermediate reduction. This raw approach precludes usage in viewing or online applications, where intermediate and large query areas will cause latency or failure of the client application.

Existing MS storage and retrieval methods do not provide a mechanism for realtime inclusion of point metadata. Several recent data processing algorithms segment MS data into component signals [7, 8]. Segmentation algorithms attempt to delineate signals first into extracted ion chromatograms (XICs), then combine them into isotopic envelopes (illustrated as neutromer traces and chargite distribution traces the unambiguous nomenclature proposed in [9]). While at least two static, XML-based data formats have been proposed to store point metadata (featureXML [10] and peakML [11]), to date no system has been proposed that can store signal segmentations as they are delineated, preventing real-time acquisition and modification of point metadata.

Current methods for MS storage and retrieval do not accommodate the following use cases:

- Processing of the entire raw file through queries of adjacent regions, such as in file format conversion and peak picking.
- Accessing large m/z, RT windows of data, such as during algorithmic processing and to supply viewers capable of zooming out to wide views.
- Querying local m/z, RT windows of the file for zoomed in views and data processing algorithms such as XIC extraction or precursor mass and intensity calculation.
- Assigning, storing, and retrieving segmentation IDs associated with each point for development, testing, and evaluation of XIC extraction and envelope clustering (feature detection) algorithms.
- Repeatedly querying adjacent data regions, such as when scrolling left, right, up, or down in m/z or RT in an MS1 viewer such as JS-MS.

In this manuscript, we present MzTree, a novel storage and retrieval system for MS data. MzTree is comprised of a novel file format, a sophisticated data structure, and a wrapping HTTP server that enable efficient MS data storage, modification, and querying. MzTree is designed for uses that require frequent, fast, axis-agnostic queries of MS1 data. It automatically constructs a sophisticated summary of data windows to provide fast returns of data at any granularity and has a point caching system that is particularly well-suited to queries of adjoining m/z, RT windows. MzTree is the first MS data storage format that models each (m/z, RT, intensity) point individually, extended with segmentation assignment attributes. MzTree can track segmentations of any shape and size, persisting all assignments for downstream processing. MzTree utilizes optimized disk I/O storage to allow for processing files of any size without suffering performance degradation. For portability and dependency-repression, MzTree is implemented in Java 8.

## Materials and methods

### Instantiation

**Fig 1:**
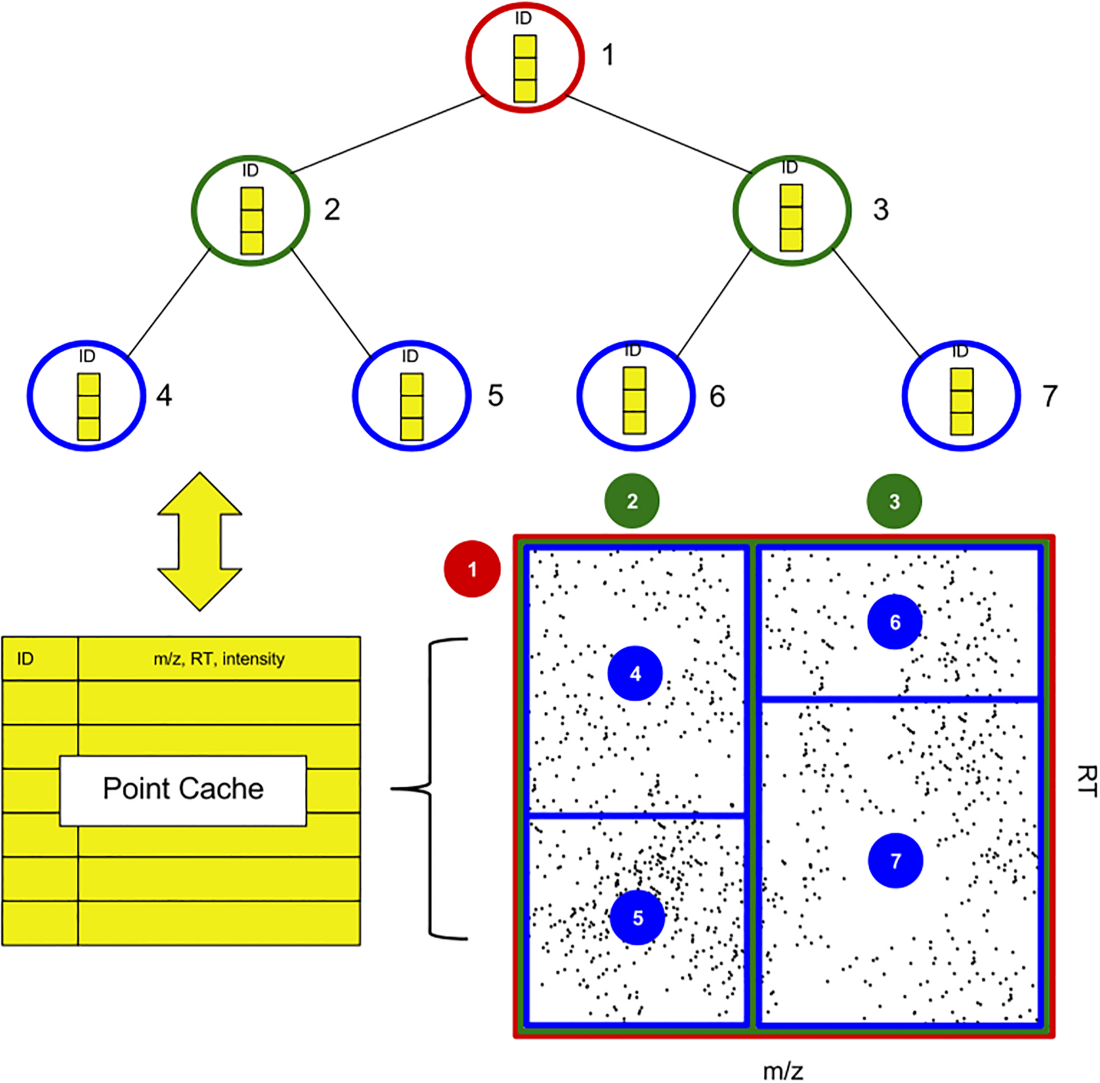
The MzTree data structure. MzTree partitions spatially-organized data by data volume, using the Sort-Tile Recursive algorithm [12]. Nodes maintain references to their points as point IDs. The point cache is a hashmap from point ID to point object (m/z, RT, intensity and XIC ID) and is the only in-memory location for MS data points.

The MzTree data structure is based on the R-Tree data structure [13]. An R-Tree is a hierarchy of data regions with each node’s region minimally encapsulating its child nodes’ data regions (bottom-right Fig 1). Leaf nodes encapsulate real data. R-Trees are queried with a two-dimensional range. The tree is traversed by expanding nodes with overlapping data regions until leaf nodes are reached.

R-Trees are designed for efficiently indexing spatial data, but do not leverage MS-specific properties, such as the tendency of MS data to cluster over specific m/zs. MzTree uses a special partitioning method that leverages these properties, resulting in fewer nodes traversed per search and, in turn, faster query response times.

**Algorithm 1** Divide: Recursively constructs an MzTree on a data set *D*_0_ given a root node *N*_0_, number of points per node *p*, sorting state *s*, and branching factor *b*

1: **procedure** divide(*D*_0_, *N*_0_, *s*, *p*, *b*)

2:  **if** *length*(*D*_0_) > *p* **then**         ▷ Non-leaf nodes only

3:   **if** *s* **then**          ▷ Sorting alternates, m/z ↔ RT

4:    *D*_0_ ← *sortByRT*(*D*_0_)

5:   **else**

6:    *D*_0_ ← *sortByMz*(*D*_0_)

7:   **end if**

8:   *l* ← *length*(*D*_0_)/*b*             ▷ Partition size *l*

9:   *i* ← 0

10:   **while** *i* < *length*(*D*_0_) **do**

11:    *D* ← *D*_0_[*i* * *l* : (*i* + 1) * *l*]            ▷ Partition

12:    *N* ← *Node*()                ▷ Child node

13:    *divide*(*D*, *N*, !*s*, *p*, *b*)          ▷ Recurse, negate *s*

14:    *addChild*(*N*_0_, *N*)

15:    *i* ← *i* + *l*

16:   **end while**

17:   *N*_0_.*pointIDs* ← *weightedStriding*(*N*_0_, *p*)

18:  **else**

19:   *N*_0_.*pointIDs* = *getPointIDs*(*D*_0_)

20:  **end if**

21: **end procedure**

22: **procedure** addChild(*N*_0_, *N*)

23:  *N*_0_.*minMz* ← *min*(*N*_0_.*minMz*, *N*.*minMz*)

24:  *N*_0_.*maxMz* ← *max*(*N*_0_.*maxMz*, *N*.*maxMz*)

25:  *N*_0_.*minRt* ← *min*(*N*_0_.*minRt*, *N*.*minRt*)

26:  *N*_0_.*maxRt* ← *max*(*N*_0_.*maxRt*, *N*.*maxRt*)

27:  *N*_0_.*children*.*append*(*N*_0_)

28: **end procedure**

Lines 3-6 of Algorithm 1 describe how MzTree partitions successive subspaces of an MS file by iteratively prioritizing either the m/z or RT dimension on each divide, resulting in an MzTree optimally balanced for any m/z, RT window query. The division of the sorted data is determined using the Sort-Tile-Recursive (STR) algorithm [12]. STR is designed to divide R-Tree partitions by point volume, not data range, ensuring full or nearly full nodes at every point in the tree.

Inputs to the procedure are a data set *D*_0_, a root node *N*_0_, number of points per node *p*, and branching factor *b*. The number of points per node *p* dictates recursion: the base case triggers when the length of *D*_0_ becomes less than *p*–the current node is deemed a leaf node and all point IDs are collected (Line 19). Non-leaf nodes proceed into the recursive block. In the recursive block, a partition size is calculated as the length of the data set over the branching factor *b* (Line 8). The data set is equally partitioned by volume and not area. For each partition, a new child node is constructed (Line 12); both the partition and child node are passed in a recursive call with the sorting flag switched (Line 13). Switching the sorting flag changes the partitioned dimension, producing the rectangular partitions illustrated by Fig 1. *Divide* is not fully tail-recursive; the current node must collect its child node after return of the recursive call. Adding a child consists of storing a reference to the child node and subsuming the child’s data bounds.

### Weighted striding

As the tree is instantiated, a process referred to as *summarization* is performed at each node (see Algorithm 1 line 17). Summarization is required in all applications that require accessing more data than can be efficiently transmitted. For example, summarization is required when an algorithm needs information regarding a large window of data to make a decision, but need not know everything about every point in the window, or when a user wishes to view the overarching layout of an entire run, but without needing the viewer to render every single point. Summarization is a novel aspect of MzTree, and occurs in two contexts: static summarization, which occurs at instantiation, and dynamic summarization, which occurs at query time. Both static and dynamic summarization are performed using a novel procedure referred to as *weighted striding*. Weighted striding is designed with three objectives:

1. **Integral samples:** Summaries must be samples of real points, not derived points. This requirement prevents the context-specific bias implicit in any derivation technique, as well as the computational overhead required to calculate derived summaries.
2. **Representative sub-samples:** Summaries must visually resemble the underlying data. This requirement increases the odds that high-level impressions to users or algorithms convey the same information conveyed by the underlying data.
3. **Deterministic sub-samples:** Samples must be collected without stochasticity. This requirement provides repeatable consistency that can be used to engineer decisions in algorithms and ensures a uniform, reproducible user experience.

**Fig 2:**
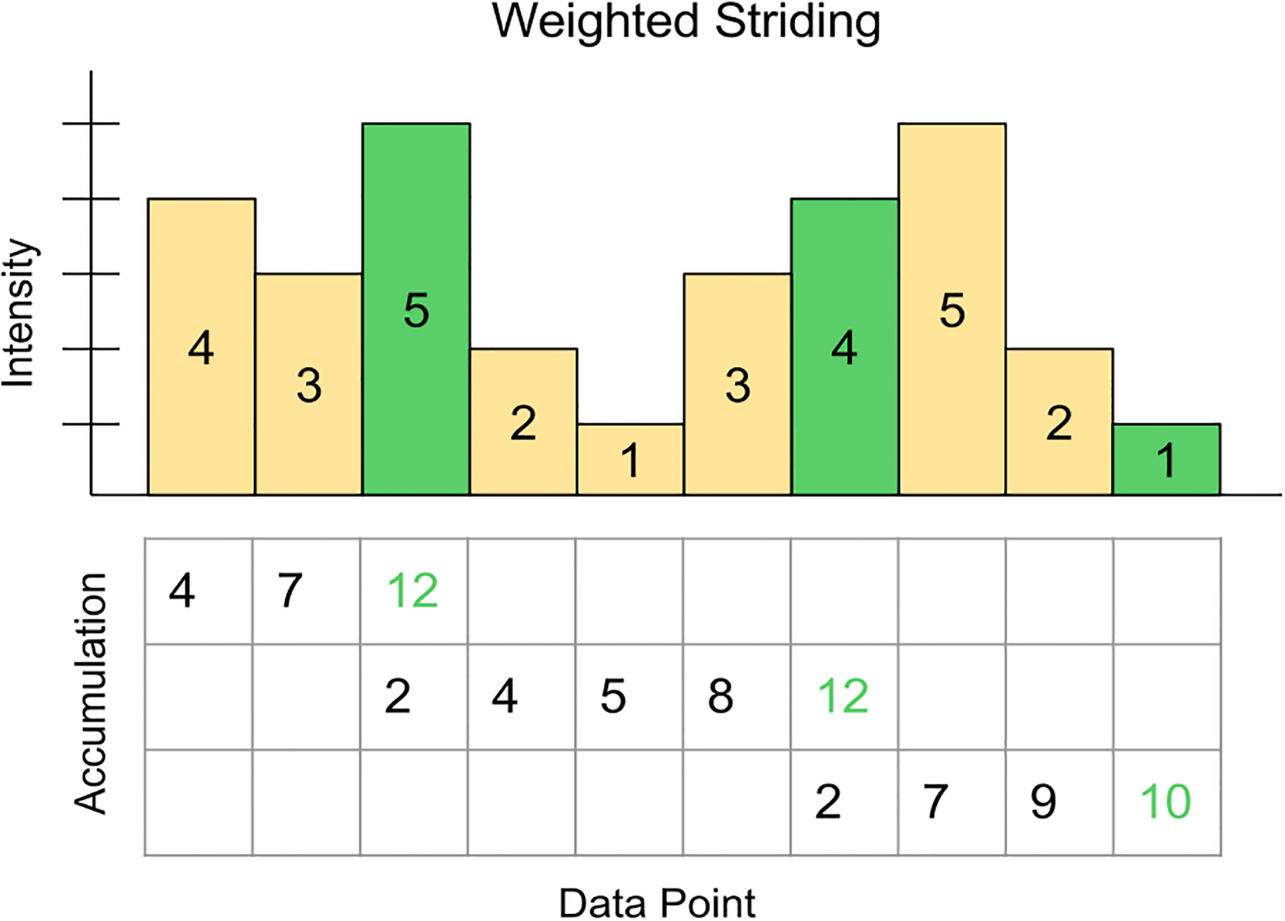
Weighted striding summarization. The weighted striding technique promotes higher intensity points while still admitting low intensity points to achieve a representative sub-sample. Shown here with10 points total, *total intensity* = 30, *stride length = 1*, *sample size = 3*, and *accumulation threshold = 10*.

Weighted striding provides point subsamples that preserve the ratio of high and low intensity points from the original data. In effect, it emulates windowed averaging without the need to derive new summarized points. It provides stochastic-like subsamples through a completely deterministic process, which is required in order to reproduce the same response to the same query. Weighted striding iterates through the list of points in a given window of data, recruiting the requested number of points for the subsample by accumulating intensity until a threshold is met (see Fig 2). With each point assignment, the process is continued with any intensity over threshold added to the next iteration. The process continues, wrapping back to the start of the array if necessary, until the requested number of points in the subsample are recruited. Fig 2 depicts the weighted striding technique with a stride length of 1 for demonstration purposes. In practice, higher values provide more representative samples, as the multiple passes they provide over the point array provide representation of areas that might be skipped over with smaller stride lengths. Experimental experience suggests 42 to be a suitable number for the stride length. Additionally, the method does not contain any stochastic elements. Repeated queries will always receive the same points.

### Static summarization

Static summarization occurs at MzTree build time to minimize response time for each query. The MzTree’s hierarchical structure consists of layers of nodes containing point IDs corresponding to the weighted striding of contiguous areas of the data set in increasing granularity, with leaf nodes consisting of the actual points (see Fig 1). At each hierarchical layer of the MzTree, a summarization is compiled and stored. Summarization does not require significant storage resources, as only the IDs of the subsample are duplicated.

### Dynamic summarization

During use, MzTree responds to queries consisting of a data window described by m/z and RT bounds, as well as a requested number of points, specific to the application. To respond to the query, the MzTree nodes are traversed breadth-first until sufficient summary nodes are reached to provide the requested number of points, or, if insufficient points exist in the queried region, until leaf nodes are reached and all points within the bounds are returned. Because it is unlikely that the number of points in the union of pertinant summarization nodes exactly equals the number of points requested in the query, weighted striding is performed on the raw result to create a subsample of points of appropriate cardinality.

### Memory management

XML-based MS data formats with sizes up to dozens of gigabytes, even up to 50 gigabytes are routinely encountered [14], presenting an ever-increasing challenge for efficient data storage and processing. Most MS data processing software is not prepared to manage this volume of data [14], as they require loading the entire MS dataset into main memory (RAM) to initiate analysis. MzTree is intentionally designed with a user-set memory upper bound, ensuring that a data set of any size can be processed by machines with insufficient memory to store the entire file. The memory threshold dictates what share of the MzTree is stored on the disk, and what portion can be stored in a RAM-based cache. MzTree moves data from disk into cache based on past user requests. The size of the cache and the proximity (in RT or m/z) of successive queries increases the hit rate of pre-cached data. By default, eighty percent of the JVM heap is allocated to the point cache.

### Point metadata

**Fig 3:**
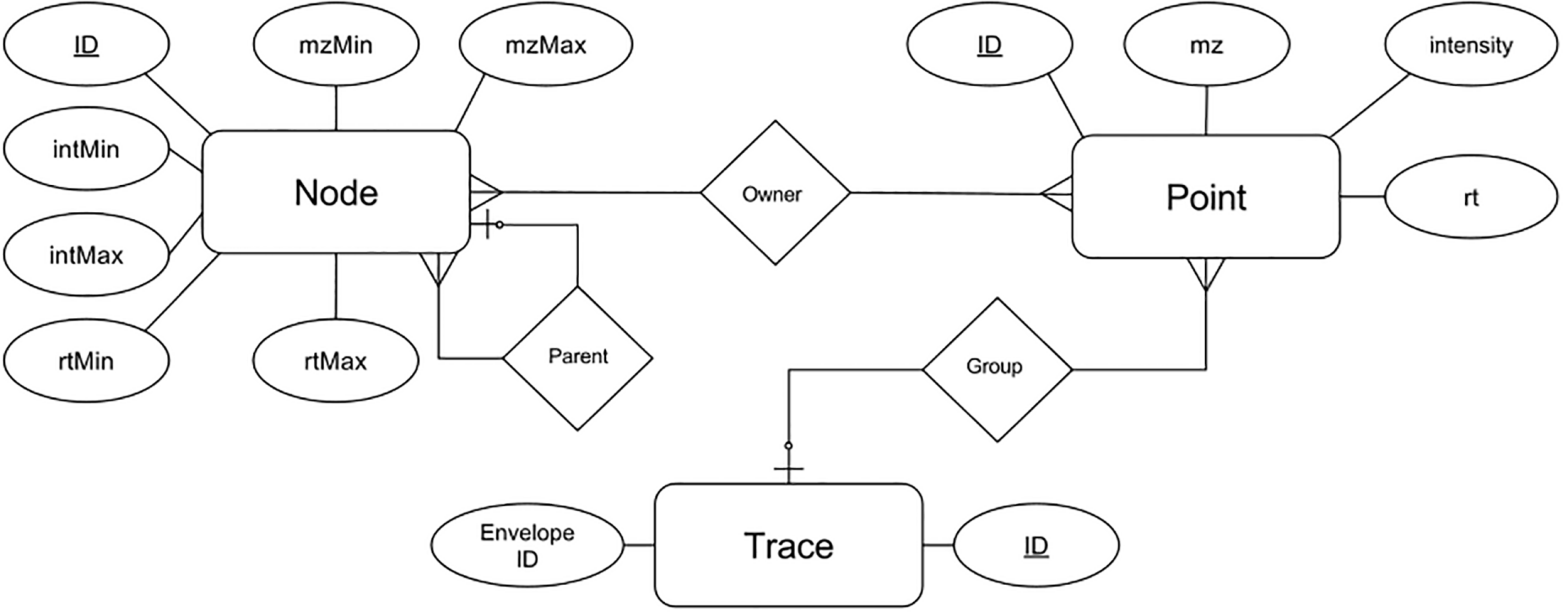
MzTree entity-relationship diagram. Entities modeled are MzTree Nodes, MS Data Points, and XICs (traces). The MzTree data store stores each entity relationally.

MzTree is a good candidate for data processing applications. MzTree models data points individually (see Fig 3), and allows the storage, retrieval, and modification of point-specific metadata, such as membership in XICs or isotopic envelopes, in real-time. MzTree’s point metadata can be used by data processing algorithms such as correspondence [15], alignment, segmentation, or feature finding. Communication with MzTree is facilitated by the HTTP protocol, making it possible to run MzTree locally or on a remote server. The API is based on JSON, making it clear and easy to use.

### File structure

Each MzTree is comprised of MS point data, MzTree node data, and point metadata. All entities are modeled relationally (see Fig 3). For rapid retrieval and real-time editing, the summarized nodes and point metadata items (such as XIC and isotopic envelope membership) are stored in an SQLite database. Because they are immutable, the point entities are stored in a custom binary format, allowing random access, providing constant time lookup as opposed to the logarithmic times required by indexing schemes.

In addition to constant time point access, the point file optimizes access to leaf nodes by physically grouping points of a leaf node. During construction of an MzTree, each leaf node’s data partition is written to the data store atomically, minimizing the number of file blocks used to store the partition, and logically storing the leaf node’s partition as a contiguous block. Given a leaf node, MzTree can navigate to its data block in constant time, and read its data partition with only a single file seek. For queries that do not require summarization, MzTree will navigate directly to the appropriate leaf nodes and return the requested data points with as few file seeks as possible. This upgrade, compared to the previous, disarranged version of the point file, presented a 3-fold improvement in query time.

### File conversion

The current MzTree implementation has three methods of instantiation corresponding to the input data. Each method requires an initial, nominal time requirement that is orders of magnitude faster than running a mass spectrometry experiment (see Results below).

The first method imports from an mzML file. Importing an mzML file begins by streaming mzML spectra data and converting it to (m/z, RT, intensity) tuples. To ease processing demands and reduce noise level, points with an intensity value below one are excluded. Each point is written to the MzTree point storage as it is read from the mzML file. After all points are imported, the hierarchical summary nodes are constructed as described above.

To facilitate data processing, MzTree can also import or export .csv files with the schema *[m/z, RT, intensity, XIC ID, isotope distribution ID]*. Each line is parsed as an MS data point, then construction of the MzTree continues identically to construction via an mzML file. MzTree can export point data of a data range to a CSV of this format. Supporting CSV import/export allows MzTree to produce segmented data in an easily-accessible format for downstream processing.

Once an MzTree is instantiated, it can almost instantaneously be accessed at any time.

### Memory requirements

mzML file sizes can be as large as tens of gigabytes. As the field advances, this size is expected to trend upwards [14]. To date, most applications that provide interactive sessions with MS data are required to load the entirety of the data set into memory. In our experience, representing all points in an mzML file in tuple form requires around 3x the memory of native mzML. The required memory for conversion simply isn’t available on most desktops. Constructing an R-Tree requires the entire data set to be sorted along a single axis before partitioning at each recursive step. By building the first level of the tree based on the RT dimension, MzTree avoids memory limitations by reducing load requirements to a single scan instead of the entire mzML file, and enabling MzTree to process an mzML file of any size. Due to memory requirements of the MzTree structure itself, at least 1GB of RAM is required for the application.

## Results and discussion

By specifically optimizing for them, MzTree outperforms existing methods on use cases of interest, including:

- Processing of the entire raw file through queries of adjacent regions, such as in file format conversion and peak picking.
- Accessing large m/z, RT windows of data, such as during algorithmic processing and to supply viewers capable of zooming out to wide views.
- Querying local m/z, RT windows of the file for zoomed in views and data processing algorithms such as XIC extraction or precursor mass and intensity calculation.
- Repeatedly querying adjacent data regions, such as when scrolling left, right, up, or down in m/z or RT in an MS1 viewer.

Here we compare MzTree to leading MS file formats most like it–mzML, mz5 [5] and mzDB [6]–in the arenas of data querying, file conversion time and size on disk. Querying comparisons are of two kinds: random and path. Random queries are a sequence of randomly generated queries over the dataset designed to measure the performance of applications that perform several successive unrelated queries; path queries are a sequence of queries that stochastically traversing the dataset from a random seed, designed to measure the performance of applications that perform several successive queries to adjacent areas. In an attempt to illustrate the effects of several optimizations, MzTree’s performance is measured with and without certain optimizations.

All experiments were completed on a Dell XPS 8900, 8-processor Intel Core i7-6700K CPU @ 4.00GHz, 256GB SSD, with Xubuntu 16.04 (except mzDB conversions), with 32GB RAM. The mzDB conversions were conducted on the same machine, but under Windows, since *pwiz-mzdb* is only available for Windows.

To perform tests two Java code stubs were written to directly access MzTree’s query and conversion functions without HTTP server related overhead. Proteowizard [16] version 3.0.9870 presents the *msaccess* and *msconvert* modules for querying and converting, respectively, mzml and mz5. The mzDB group provides publicly available modules, *mzdb-access* version 0.5.0 and *pwiz-mzdb* version 0.9.9, for querying mzDB and converting from Thermo RAW to mzDB, respectively. The latest versions of these modules can be found at github.com/mzdb/mzdb-access. *pwiz-mzdb* is only available for Windows, thus RAW to mzDB was performed on the Windows partition on the same drive of the same machine used for all other tests.

Sixteen public .mzML and raw files were used for the analysis. Though these datasets were pulled from ProteomeXchange [17] for convenience, results should generalize to any complex LC-MS experimental data including, for example, lipid and metabolomics data. Identifiers for each dataset as well as detailed results for random querying, path querying, conversion time and size on disk are available in the supplementary data.

### Queries of large regions

All MS data processing situations involve a limited amount of RAM, either imposed by the platform used (such as a browser’s maximal number of points renderable, or a system’s available RAM), yet none of the methods discussed here have the ability to return information about a data region or entire file when the area of interest includes too many points to store in memory.

**Fig 4:**
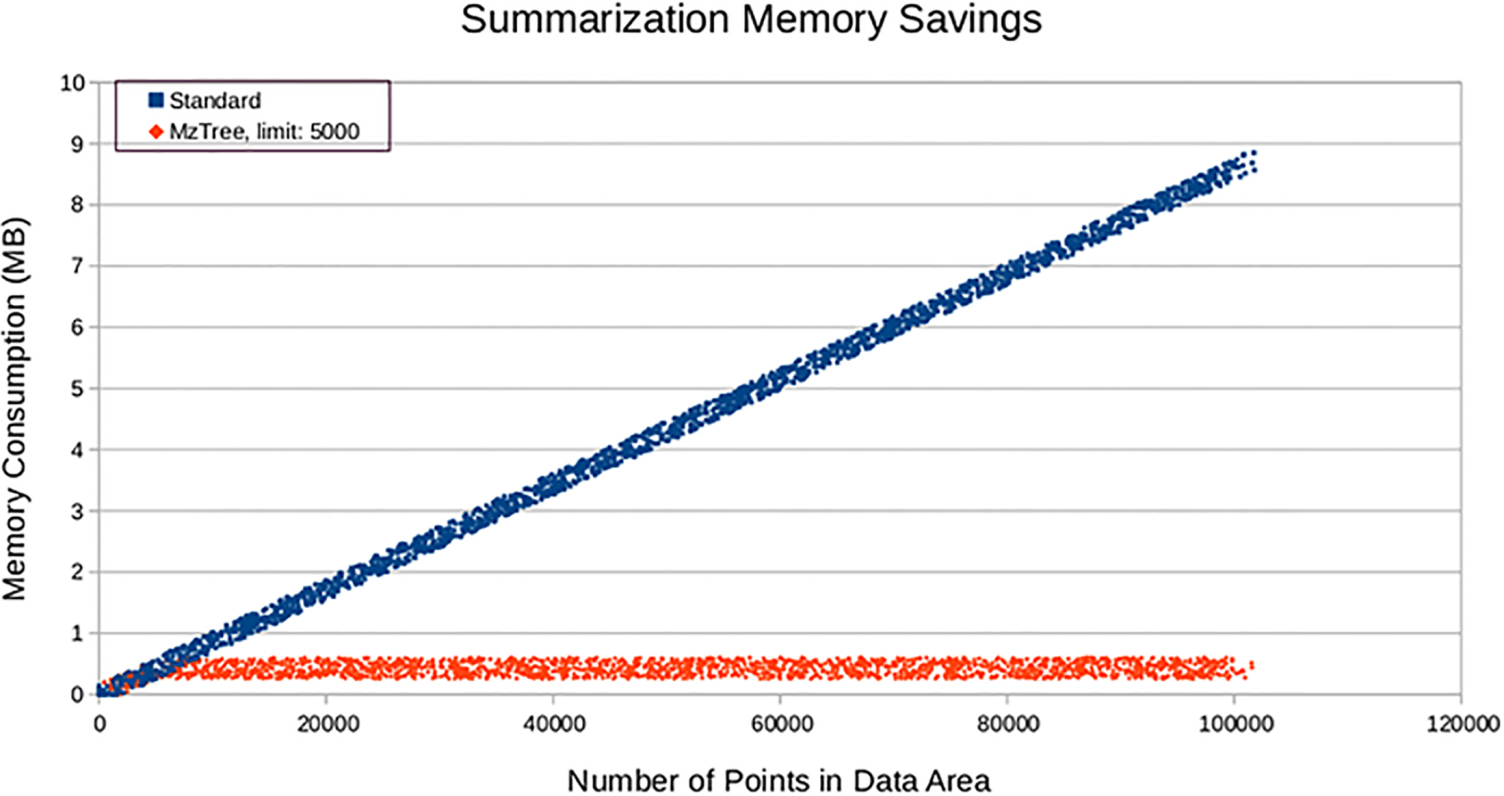
Summarization memory savings. Of all methods discussed in this paper, MzTree is the only one that can handle data sets that require more RAM than the application provides. MzTree’s summarization feature (shown here with limit set to 5,000 points) allows users to work with arbitrarily large data sets even in situations where available RAM is less than that required to hold the unsummarized set of returned points, such as system memory limits or visualization rendering limits.

MzTree’s summarization technique allows the user to retrieve information about any file or any region in any file no matter how large that region may be (see Fig 4. This allows the user to use MzTree as a data source for algorithmic data processing or visualization on any MS data set, not just those that fit entirely in memory.

### Non-adjacent queries in either dimension

**Fig 5:**
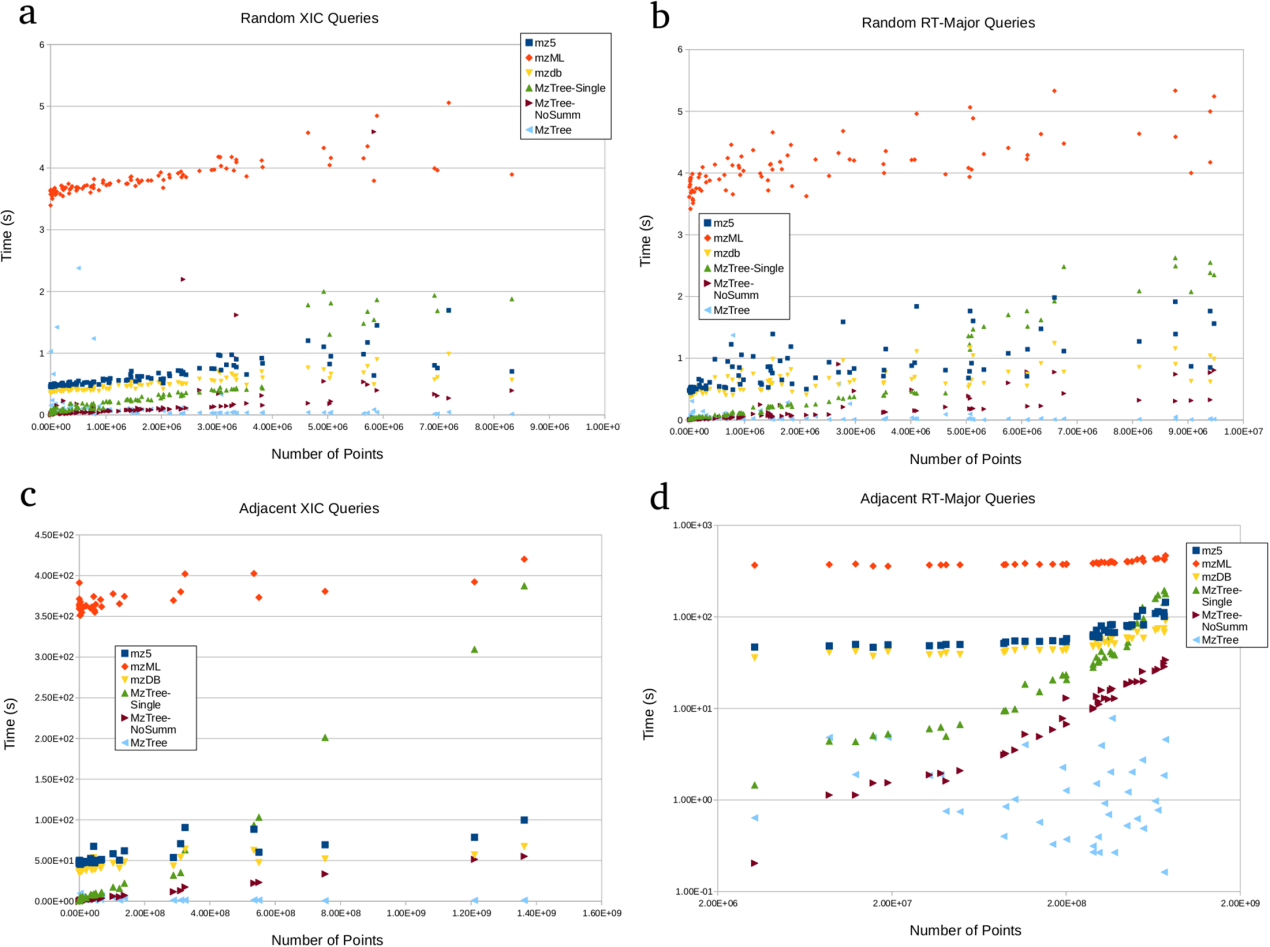
Query response times. (a) Query times from 100 random queries from non-adjacent regions where query RT height < m/z width. MzTree is able to retrieve m/z-major data regions faster than any other analyzed method. Without summarization (that is, returning all points in the region), MzTree retrieves all points faster than other methods with only a few exceptions. (b) Query times from 100 random queries from non-adjacent regions where query RT height > m/z width. Mz tree is able to retrieve RT-major data regions at least as fast as other methods on small queries, and much faster on larger queries. (c) Query times from 40 paths through adjacent regions with 100 queries each where query RT height < m/z width. While mzML outperforms mz5 and mzDB, MzTree outperforms all methods on realistically-sized (¡multiple GB) queries. In addition to the standard MzTree and MzTree without summarization, a single-instance MzTree is shown to quantify the benefits of the point cache (set to 80% of the default JVM heap), a feature other methods do not have. (d) Query times from 40 paths through adjacent regions with 100 queries each where query RT height > m/z width. While mzML outperforms mz5 and mzDB, MzTree outperforms all methods on realistically-sized (¡multiple GB) queries. In addition to the standard MzTree and MzTree without summarization, a single-instance MzTree is shown to quantify the benefits of the point cache (set to 80% of the default JVM heap), a feature other methods do not have.

Most current data processing queries use m/z-major data regions, where the RT height of the query bounds exceeds the m/z width. In these types of queries, MzTree retrieves points in less time than competing methods (see Fig 5(a)). This performance holds whether or not summarization is used.

In some use cases, such as alternative data processing techniques and visualization scenarios, the user is more interested in RT-major data regions, where the m/z width of the query bounds exceeds the RT height. Because of axis-agnostic design, MzTree is comparably fast in both m/z- and RT-major queries, where competing methods perform relatively worse than in m/z-major queries (see Fig 5(b)). MzTree’s improved performance is due to axis-agnostic design, as both mz5 [5] and mzDB [6] are optimized for m/z-centric queries.

### Adjacent queries in either dimension

Use cases such as total coverage data processing or visualization will generate many queries to adjacent regions of data as the algorithm/user processes the next portion of the file. MzTree’s point cache is specifically designed as an optimization for this use case. Point caching retains the points from the previous query for reuse when the next query region overlaps the previous.

For this section, 25 different sequences of 100 queries were generated by simulating user navigation through the 301M point dataset. Paths were generated by mutating a data range with a random jump to either zoom-in, zoom-out, move up in RT, down in RT, up in m/z, or down in m/z. Each of the resulting 25 query paths were subjected to each file format, and the overall number of points and overall execution time per path were recorded.

For both RT-major queries (see Fig 5(c)) and m/z-major queries (see Fig 5(d)), MzTree outperforms other methods on queries over several orders of magnitude in size.

### File conversion

**Fig 6:**
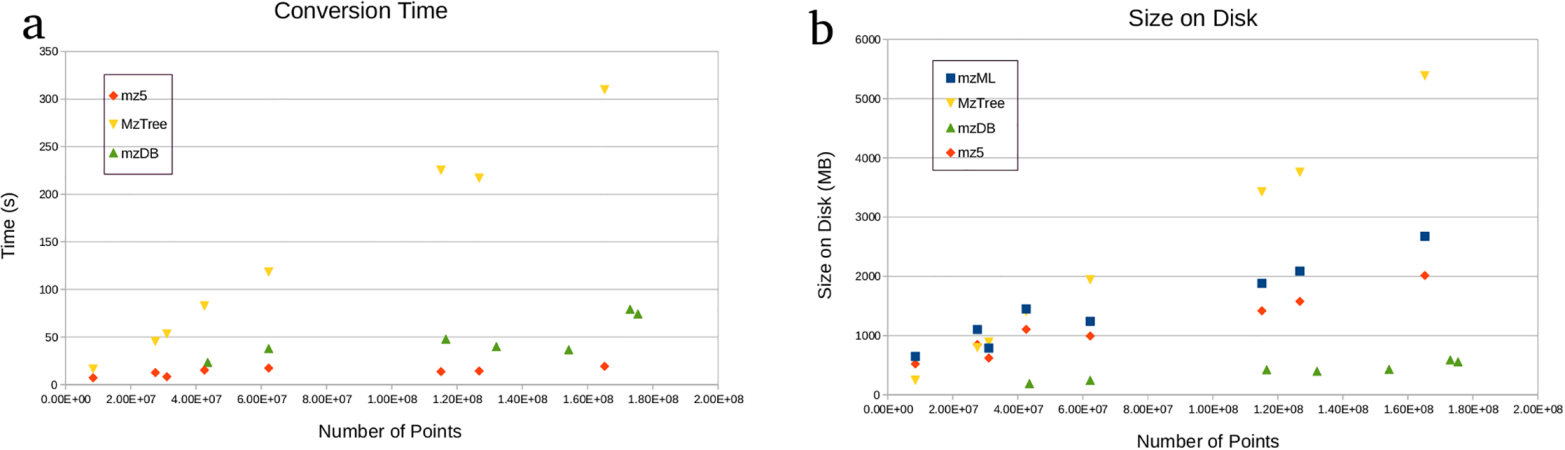
Footprint of data structures as function of number of points. The MzTree grows in time and space roughly linearly with the number of points, but the one-time conversion takes far less time than an MS experiment and provides optimized savings over multiple queries. (a) Time required to convert from mzML to MzTree. (b) Space required for an MzTree.

Conversion times from mzML to each file format are displayed in Fig 6(a). Note that because mzDB is not currently able to convert from mzML, times reported for mzDB are from Thermo RAW directly to mzDB using the *pwiz-mzdb* version 0.9.9 module from Proteowizard [16] version 3.0.9870. For the other methods, RAW files were converted to mzML using Proteowizard’s *msaccess* and *msconvert* modules.

The resulting sizes of each data structure on disk after conversion are reported in Fig 6(b). The size of the MzTree grows roughly linearly with the number of points.

Part of MzTree’s optimization consists of a data structure intentionally designed for the use cases described above. Though there is an overhead cost associated with file conversion, the time requirement is orders of magnitude smaller than the actual MS experiment, while the space requirement scales roughly linearly with the number of points. As a one-time cost, the conversion time and space amortizes well in cases where multiple queries will occur on one file.

It should be noted that MzTree is not designed for every MS data processing use case. The version described here requires a binary format of fixed size, and cannot be dynamically expanded to retain additional information, such as MS/MS spectra, imzML format, or drift time, although the system can be expanded programmatically to create a new file time to accommodate these and any other use cases. For now, users with differing use cases than those described above are advised to use mzDB. Future work on MzTree will include the ability to dynamically expand file types.

## Conclusion

Unbiased MS data processing and viewing requires axis-agnostic querying and modification abilities. Current methods for MS data storage are RT-centric, precluding axis agnostic queries and create a static data structure that does not allow the addition of point metadata for signal segmentation.

MzTree provides an optimized solution for repetitive queries of m/z, RT windows of data suitable for axis agnostic data processing algorithms and efficient MS viewers by leveraging a point tree with levels alternatively sorted by RT or m/z, and with nodes in the tree corresponding to rectangular regions with similar cardinality. The subset of points in each node is determined by a novel algorithm for point summarization called weighted striding, which preserves signal shape while providing an upper bound on memory usage across data segments from local neighborhoods to entire MS files. MzTree includes an HTTP server as one possible implementation of the query API.

In comparison to existing MS data storage methods, MzTree performs faster in servicing both random and sequential queries, regardless of the major axis of the query. It features reasonable conversion times, and is capable of maintaining memory limitations on MS files of any size. It is freely available under GPL 3.0 at https://github.com/optimusmoose/MZTree.

## Supporting information

S1 File Supplementary text. Listing sources of data used in analysis.
(DOCX)

S2 File Supplementary data. Conversion time and size on disk results for all formats.
(ODS)

S3 File Supplementary data. RT-major adjacent query results.
(ODS)

S4 File Supplementary data. RT-major random query results.
(ODS)

S5 File Supplementary data. XIC adjacent query results.
(ODS)

S6 File Supplementary data. XIC random query results.
(ODS)

S7 File Supplementary data. Simulated summarization results.
(ODS)
