# Supplementary material for: Fast, axis-agnostic, dynamically summarized storage and retrieval for mass spectrometry data
Source: PLoS One. 2017 Nov 15;12(11):e0188059. doi: 10.1371/journal.pone.0188059 (PMC5687738; doi:10.1371/journal.pone.0188059)
Supplement: S1 File — Listing sources of data used in analysis. (DOCX) [file pone.0188059.s001.docx]

Dataset Sources

Datasets used in the conversion time and size on disk comparisons were downloaded from ProteomeXchange and can be found at <http://www.proteomexchange.org/> under the given name in the repository given by the proteomeXchange ID.

The following files were used in testing conversion time and size on disk between *.mzML, .mz5 and* MzTree:

*CHPP_SDS_3002* **- PXD004785**

*GRAIN_DEVELOPMENT_Z71_3* **PXD004720**

*STEM_12* **- PXD004720**

*18185_REP2_4pmol_UPS2_IDA_1* **- PXD001587**

*POLLEN_2* **- PXD004720**

*Sheppard_Werner_RNAPORF145_09* **- PXD003282**

*Sheppard_Werner_RNAPORF145_06* **- PXD003282**

*Sheppard_Werner_RNAPORF145_03* **- PXD003282**

The following *.raw* files were used in testing mzDB’s conversion time and size on disk:

*140924_11.raw* **- PXD004599**

*140924_12.raw* **- PXD004599**

*D1_Control4_TechRep_1.RAW* **- PXD004499**

*PaMA150528_J4-3_Q1468.raw* **- PXD004313**

*PaMA150528_J7-2_Q1475.raw* **- PXD004313**

*PaMA150528_J8-3_Q1262_150713163831.raw* **- PXD004313**

*PaMA150528_JA5-4_Q1255.raw* **- PXD004313**

*Singer_BGWC_8plex_04_9May13_Methow_13-02-13.raw* **- PXD004204**

The dataset used to perform query tests can be found in Thermo RAW, mascot and mzDB format at <http://proline.profiproteomics.fr/download/> under the name OEMMA_121101_61b.*

Supplementary Data

Included are six spreadsheets containing the query and conversion results summarized in the manuscript figures.

- **Supplementary Data S2.ods:** conversion time and size on disk results for all formats
- **Supplementary Data S3.ods:** rtmajor adjacent query results
- **Supplementary Data S4.ods:** rtmajor random query results
- **Supplementary Data S5.ods:** xic adjacent query results
- **Supplementary Data S6.ods:** xic random query results
- **Supplementary Data S7.ods**: simulated summarization savings, with jitter
